# Distributed Poloidal Magnetic Field Measurement in Tokamaks Using Polarization-Sensitive Reflectometric Fiber Optic Sensor

**DOI:** 10.3390/s23135923

**Published:** 2023-06-26

**Authors:** Prasad Dandu, Andrei Gusarov, Willem Leysen, Perry Beaumont, Marc Wuilpart, JET Contributors

**Affiliations:** 1 Department of Electromagnetism and Telecommunications, University of Mons, 7000 Mons, Belgium; 2 Belgian Nuclear Research Center SCK-CEN, 2400 Mol, Belgium; 3 JET, CCFE, Culham Science Center Abingdon, Oxon OX14 3DB, UK

**Keywords:** distributed magnetic field measurement, optical fiber sensor, polarization-sensitive reflectometry, JET magnetic field, tokamak, poloidal magnetic field measurement

## Abstract

Determination of the poloidal magnetic field distribution in tokamaks is of prime importance for the successful operation of tokamaks. In this paper, we propose a polarization-sensitive reflectometry-based optical fiber sensor for measuring the spatial distribution of the poloidal magnetic field in tokamaks. The measurement method exploits the Rayleigh backscattering and Faraday magneto-optic effect in optical fibers. The former is an intrinsic property of optical fibers and enables distributed polarization measurements, while the latter arises in the presence of a magnetic field parallel to the optical fiber axis and rotates the polarization state of the light. When an optical fiber is looped around a toroidal section of the vacuum vessel, the local polarization rotation of the light is proportional to the local poloidal magnetic field in the tokamak. The proposed method is discussed theoretically and experimentally using the results from JET. The obtained magnetic field measurement shows a good agreement with that of the internal discrete coils. A potential solution to recover the magnetic field data from the noise-affected region of the optical measurement is proposed and is demonstrated through simulations using the JET magnetic field configuration.

## 1. Introduction

The measurement of the poloidal magnetic field distribution in tokamak devices is crucial for understanding the confinement and stability of the plasma [1]. Conventionally, magnetic field measurements are made by using inductive sensors like Rogowski and pickup coils [2]. However, these sensors may not operate appropriately in future burning plasma installations, such as ITER and DEMO, due to the strong radiation and steady-state plasma operation [3,4,5]. Fiber optic current sensors (FOCSs), on the other hand, have the potential to operate effectively in steady-state conditions. However, their performance in the harsh radiation environment of future tokamaks, such as ITER, may be affected by radiation-induced attenuation (RIA) and radiation-induced changes in the Verdet constant of the sensing fiber. Preliminary research results on these aspects suggest that the performance of Faraday-effect-based optical fiber sensors is not significantly impacted in the harsh radiation environment expected in ITER [6]. Therefore, FOCSs are being considered for plasma current measurement in ITER [7,8]. Using a polarization-sensitive reflectometry (PSR)-based interrogation technique [9], an optical fiber could be used to measure both the plasma current and spatial distribution of the poloidal magnetic field in tokamaks. The plasma current measurement ability of the PSR-based optical fiber sensor was successfully demonstrated by using an experiment carried out at the Tore Supra (now WEST) tokamak [10,11]. This paper addresses the distributed poloidal magnetic field measurement ability of the PSR-based optical fiber sensor. The merit of the proposed sensor lies in the fact that a single optical fiber sensor placed around a toroidal section of the tokamak can provide the poloidal magnetic field information that comes from hundreds of discrete sensors and help to determine the actual poloidal magnetic field profile. Such an ability is unparalleled by any other conventional tokamak magnetic field measuring sensors.

The idea [12] of achieving distributed magnetic field measurement based on the Faraday magneto-optic effect is as old as the first polarization-sensitive reflectometer, i.e., the polarization-sensitive optical time domain reflectometer (POTDR) [13]. However, until recently [14,15], there was no true demonstration of the Faraday-effect-based distributed magnetic field measurement. In [14,15], magnetic field measurement was carried out in a magnetic resonance imaging (MRI) scanner using a polarization-sensitive optical frequency domain reflectometer (POFDR). The measurement method was based on the complete measurement of the state of polarization (SOP) of the backscattered light, i.e., the sensing fiber has to be analyzed by a few input SOP orientations. In this paper, we propose a different method for Faraday magneto-optic effect-based distributed magnetic field measurement in tokamaks. The proposed method uses a simpler PSR. Unlike the aforementioned method [14,15], a single polarization axis analysis is sufficient, thanks to the use of spun sensing fiber [16]. Polarization-sensitive photon counting OTDR (ν-OTDR) [17], referred to as ν-POTDR, was used as the PSR in this study owing to its relative cost-effectiveness. However, it is worth noting that a POFDR could also be utilized. It is also worth noting that, in general, distributed magnetic field measurement can also be achieved based on the principle of magnetostriction [18,19]. However, in the context of tokamaks, the radiation environment and the requirement for a flexible sensing element that can be looped around a section of the vacuum vessel make the Faraday-effect-based magnetic field sensing more suitable for tokamak applications [20].

The rest of the paper is organized as follows. In Section 2, the proposed measurement method is theoretically detailed, and mathematical modeling of the sensor is provided and validated by the experimental data from Joint European Torus (JET) in Section 3. Later, in Section 4, a potential solution to recover the magnetic field information lost due to the reflectometer’s noise is proposed and is demonstrated through simulations using the JET magnetic field configuration. Finally, concluding remarks are provided in Section 5.

## 2. Polarization-Sensitive Reflectometry for Distributed Magnetic Field Measurement: Theory and Sensor Modeling

**Figure 1:**
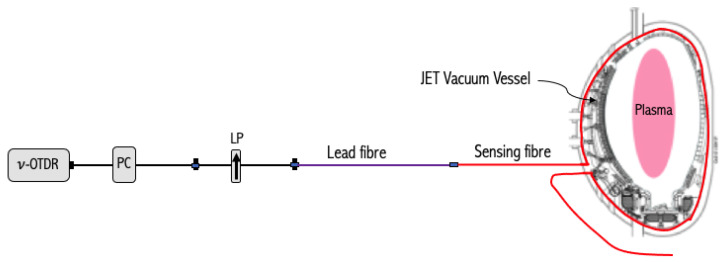
Schematic of the experimental setup of the ν-POTDR-based optical fiber sensor used to measure distributed poloidal magnetic field at JET; PC: polarization controller, LP: linear polarizer.

The schematic of the ν-POTDR experimental arrangement designed for distributed poloidal magnetic field measurement at JET [21] is shown in Figure 1. The PC placed after the ν-OTDR is used to maximize the Rayleigh backscattered signal power. It should be noted that the Aurea technology PICOXEA is the ν-OTDR used in these experiments. A lead fiber, approximately 100 meters in length, is utilized to establish a connection between the sensing fiber and the ν-POTDR interrogation unit located in the JET J1D instrumentation area. The sensing fiber is looped around a toroidal section of the JET Vacuum Vessel (VV) in Octant 3. In the present study, the ν-OTDR is operated at a wavelength of 1625 nm. The choice of this operating wavelength is primarily due to the practical limitations. However, it is worth noting that the measurement could be optimized by operating at 1310 nm, where both the Rayleigh backscattered light power and the Verdet constant (see Equation (1)) increase.

The operating principle of the measurement setup is as follows. The light pulses launched from the ν-OTDR are polarized as they pass through the linear polarizer (LP) before being subsequently launched into the lead fiber. As the polarized light propagates down the fiber, a part of it is continuously Rayleigh backscattered and reaches the reflectometer’s detector after passing through the linear polarizer again. The role of the linear polarizer in the backward propagation of the light is to convert the state of polarization (SOP) variations of the light pulse to the power fluctuation seen on the PSR trace. The PSR-based distributed magnetic field measurement relies on tracking the SOP evolution in the sensing fiber subjected to the Faraday magneto-optic effect generated by the magnetic field to be measured.

**Figure 2:**
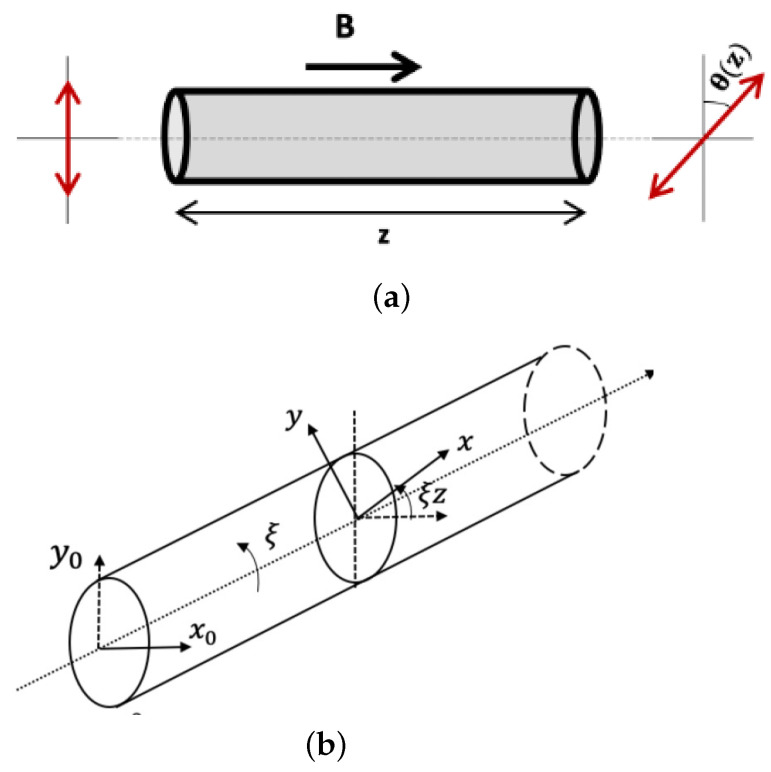
(**a**) Faraday effect in (spun or unspun) optical fiber; (**b**) schematic of spun fiber.

The Faraday magneto-optic effect or Faraday effect is a phenomenon observed in optical fibers in the presence of a magnetic field parallel to the light propagation. The action of the Faraday effect is to rotate the plane of the linear SOP propagating in the fiber [22,23]; see Figure 2a. The resulting SOP rotation, termed Faraday rotation, obtained over a distance *z* is proportional to the integral of the magnetic field induction along the fiber length [24,25]:

(1) $$\theta \left(z\right)=V\int_{0}^{z}B_{z}\left(l\right)dl$$

where $B_{z}\left(l\right)=B\left(l\right)\cos\varphi \left(l\right)$ is the amplitude of the axial magnetic field induction at a distance *l* along the fiber and is proportional to the axial magnetic field $H_{z}\left(l\right)=\frac{B_{z}\left(l\right)}{\mu_{0}}$ ($\mu_{0}$ is the vacuum permeability), φ(l) is the local angle between the magnetic field induction vector and the light propagation direction, dl is the differential length and *V* is a material constant called the Verdet constant. For a single-mode silica fiber operating at a wavelength of 1625 nm, V≈ 0.484 rad T${}^{-1}$ m${}^{-1}$ [26]. The Faraday effect is a non-reciprocal effect—orientation of the SOP rotation is the same for the forward and backward direction of light propagation, meaning that the rotation is doubled after the round-trip propagation [27,28]. The non-reciprocal nature of the Faraday effect is crucial for the Faraday-effect-based distributed magnetic field measurements [12,14,15]. The Faraday effect in single-mode silica fibers is quite weak, especially at higher wavelengths [26,29,30]. Consequently, the relatively small SOP rotation resulting from the Faraday effect is swamped by the intrinsic linear birefringence inherent to standard single-mode fibers (SMFs) [31,32]. At first glance, it might appear that a low-birefringence (lo-bi) fiber could be used as the sensing fiber. However, a lo-bi fiber, while having a very low intrinsic birefringence, is quite sensitive to external perturbations, which are pretty much inevitable in any polarimetric sensing application due to the sensitivity of the polarization of light to most of the external stimuli present in any sensing environment [33,34]. Nevertheless, a special type of fiber called spun fiber [35] is commonly used in Faraday magneto-optic effect-based sensing applications because of its ability to effectively reduce the influence of perturbing effects [16,36,37]. A spun fiber is formed by spinning the standard single-mode fiber preform during the fiber drawing process—precursor birefringence axes are rotated at a rate proportional to the spin rate [38,39]; see Figure 2b. Therefore, the precursor birefringence Δβ and spin rate ξ are the two important parameters that control the performance of the spun sensing fiber [40,41,42].

Before analyzing the experimental data, it is instructive to theoretically model the measurement principle and deduce an analytical equation, which helps in interpreting the experimental traces. In the sensor modeling, the spun sensing fiber is considered as a concatenation of *n* uniform-spun fiber elements of small length dz, over which the spun fiber parameters Δβ and ξ and the magnetic field can be considered constant. It should be noted that in this modeling, the perturbing effects such as fiber bending, twisting and ambient temperature acting on the spun sensing fiber are neglected. A theoretical analysis of the perturbing effects in the context of ITER tokamak [43] suggests that by properly choosing the Δβ and ξ of the spun sensing fiber, these effects can be ignored, and the required measurement accuracy can be achieved.

In modeling the spun sensing fiber, we used the Jones retarder and rotator pair equivalent representation of an optical element without polarization-dependent losses [44,45,46]. Therefore, the Jones matrix of the ith spun fiber element, subjected to the magnetic field, can be expressed as [47]:

(2) $$M_{i}^{k}=\Omega_{i}^{k}R_{i}^{k}$$

where i=1,⋯,n; *k* denotes forward (when *k* replaced by *f*) and backward (when *k* replaced by *b*) light propagation directions and $R_{i}$ and $\Omega_{i}$ are the Jones matrices that represent the linear retardation and rotation action of the ith spun fiber element, respectively, and are expressed as [47]:

(3) $$R_{i}^{k}=\left[\begin{array}{ccc} \cos\frac{R_{i}^{k}}{2}+j\sin\frac{R_{i}^{k}}{2}\cos2\phi_{i}^{k} & \; & j\sin\frac{R_{i}^{k}}{2}\sin2\phi_{i}^{k}\\ j\sin\frac{R_{i}^{k}}{2}\sin2\phi_{i}^{k} & \; & \cos\frac{R_{i}^{k}}{2}-j\sin\frac{R_{i}^{k}}{2}\cos2\phi_{i}^{k} \end{array}\right]$$

(4) $$\Omega_{i}^{k}=\left[\begin{array}{cc} \cos\Omega_{i}^{k} & -\sin\Omega_{i}^{k}\\ \sin\Omega_{i}^{k} & \cos\Omega_{i}^{k} \end{array}\right]$$

with

(5) $$R_{i}^{k}=2\arcsin\left(\frac{\Delta \beta }{2\Delta_{i}^{k}}\sin\left(\Delta_{i}^{k}dz\right)\right)$$

(6) $$\Omega_{i}^{k}=\xi^{k}dz+\arctan\left(\frac{-(\xi^{k}-\rho_{i})}{\Delta_{i}^{k}}\tan\left(\Delta_{i}^{k}dz\right)\right)$$

(7) $$\phi_{i}^{k}=\frac{\xi^{k}dz-\Omega_{i}^{k}}{2}+q_{i}^{k}$$

(8) $$\Delta_{i}^{k}=\sqrt{{\left(\frac{\Delta \beta }{2}\right)}^{2}+{\left(\xi^{k}-\rho_{i}\right)}^{2}}$$

where $\xi^{f}=-\xi^{b}=\xi$ due to the reciprocal nature of the spinning effect-induced circular birefringence; $q_{i}^{k}$ is the initial orientation of the fast axis, with reference to the vertical, at the beginning of the fiber section, i.e., $q_{i}^{f}=q_{0}+\left(i-1\right)\xi dz$ and $q_{i}^{b}=q_{0}+i\xi dz$ ($q_{0}$ is the initial orientation of the fast at the beginning of the fiber); $2\rho_{i}$ = $2VB_{i}$ is the local Faraday-effect-induced circular birefringence and $2\Delta_{i}^{f(b)}$ is the local elliptical birefringence in the forward (backward) light propagation direction.

For brevity of the analysis, let us simplify Equation (2) based on the choice of the spun sensing fiber parameters. In general, for better sensitivity of the magnetic field, it is preferred to use a spun fiber with Δβ≪ξ; the experimental results reported in this paper are based on a spun fiber with $\frac{\xi }{\Delta \beta }\approx$ 100. For Δβ≪ξ, we have:

(9) $$\Delta_{i}^{k}\approx |\xi^{k}-\rho_{i}|$$

(10) $$R_{i}^{k}\approx 0$$

(11) $$\Omega_{i}^{k}\approx \rho_{i}dz$$

Substituting Equations (9)–(11) in Equations (3) and (4) and then in Equation (2), we get

(12) $$M_{i}^{k}=\left[\begin{array}{cc} \cos\theta_{i} & -\sin\theta_{i}\\ \sin\theta_{i} & \cos\theta_{i} \end{array}\right]$$

where

(13) $$\theta_{i}=\rho_{i}dz$$

is the local Faraday rotation. The above equation indicates that the Jones matrix of each of the spun fiber elements is a rotator with a rotation angle equal to the local Faraday rotation, $\theta_{i}$. Note that $M_{i}$ is the same for both forward and backward propagation, as Faraday rotation is independent of the light propagation direction.

According to the sensing arrangement shown in Figure 1, using the Jones formalism approach [44], the SOP of the light reaching the receiver from the ith fiber element can be expressed as

(14) $$V_{out_{i}}=M_{PC}M_{P}M_{L}^{T}M_{b}M_{m}M_{f}M_{L}V_{in}$$

where $V_{in}$ is the input SOP launched through the polarizer; $M_{L}$ is the Jones matrix of reciprocal birefringence effect that represents the effect of the lead fiber, patch cords and optical connectors between the polarizer and the sensing fiber in the forward propagation of light (as these optical components are not exposed to the magnetic field) and in the backward propagation of light, the corresponding Jones matrix is $M_{L}^{T}$ (transpose of $M_{L}$) [48,49]; $M_{m}$ is the Jones mirror matrix, the identity matrix, which represents the Rayleigh backscattering effect [50,51] and $M_{f}$ and $M_{b}$ are the Jones matrices of the spun sensing fiber, until the ith element, for the forward and backward propagation of light, respectively, [44]:

(15) $$M_{f}=M_{i}^{f}M_{i-1}^{f}\cdots M_{2}^{f}M_{1}^{f}$$

(16) $$M_{b}=M_{1}^{b}M_{1}^{b}\cdots M_{i-1}^{b}M_{i}^{b}$$

and $M_{P}$ represents the effect of the polarizer in the backward propagation. Note that $M_{L}$ is represented with a general unitary matrix $\left[\begin{array}{cc} a & b\\ -b^{*} & a^{*} \end{array}\right]$, where ${\left|a\right|}^{2}+{\left|b\right|}^{2}=1$ [52]. Similarly, the effect of the polarization controller (PC) in the backward direction $M_{PC}$ can be expressed as ${\left[\begin{array}{cc} A & B\\ -B^{*} & A^{*} \end{array}\right]}^{T}$.

For convenience, let us choose a vertical polarizer, i.e., $M_{P}=\left[\begin{array}{cc} 0 & 0\\ 0 & 1 \end{array}\right]$, and a corresponding input SOP, $V_{in}=\left[\begin{array}{c} 0\\ 1 \end{array}\right]$. The resulting output SOP is given by:

(17) $$V_{out_{i}}=\left[\begin{array}{c} V_{x}\\ V_{y} \end{array}\right]$$

where $V_{x}=-B^{*}\left(b^{2}+a^{{*}^{2}}\right)\cos\left(2\theta \left(z\right)\right)$ and $V_{y}=A^{*}\left(b^{2}+a^{{*}^{2}}\right)\cos\left(2\theta \left(z\right)\right)$, z=i.dz, i.e., $\theta \left(z\right)=\sum_{j=1}^{i}\theta_{j}$. The normalized backscattered power of the trace seen on the ν-OTDR detector is

(18) $$P_{B}\left(z\right)\propto {\left|V_{out_{i}}\right|}^{2}=|V_{x}{|}^{2}+|V_{y}{|}^{2}=\left[|-B^{*}\left(b^{2}+a^{{*}^{2}}\right){|}^{2}+|A^{*}\left(b^{2}+a^{{*}^{2}}\right){|}^{2}\right]\cos^{2}\left(2\theta \left(z\right)\right)$$

The term in [] acts as a scaling factor and does not affect the spatial frequency of the backscattered power, which contains the information on the magnetic field distribution. Consequently, the normalized backscattered power can be expressed as:

(19) $$P_{B}\left(z\right)=\cos^{2}\left(2\theta \left(z\right)\right)$$

To facilitate the local magnetic field extraction, Equation (19) can be rewritten as:

(20) $$P_{fit}\left(z\right)=\cos^{2}\left(2\theta_{i}+\varphi \left(z\right)\right)=\cos^{2}\left(2\rho_{i}dz+\varphi \left(z\right)\right)$$

where φ(z) is the accumulated rotation until z−dz; recall that dz is the length of the local section of the fiber along which the magnetic field can be considered constant. The local magnetic field measurement can be achieved from the knowledge of $\rho_{i}$, i.e.,

(21) $$B_{i}=\frac{\rho_{i}}{V}$$

The process of extracting the local magnetic field can be summarized as follows. The experimentally obtained PSR trace from the sensing region has to be normalized first, which in the ideal scenario should follow Equation (19). Later, the obtained normalized trace is locally fitted with Equation (20) to find the locally best-fitted $\rho_{i}$, which gives information on the strength of the local axial magnetic field induction $B_{i}$; see Equation (21). The best local fit is determined based on the least mean square error (LMSE) [53], by sweeping ρ and ϕ over the chosen range. The range of ρ should be higher than the value of ρ that corresponds to the maximum magnetic field induction to be measured, while the range of φ is chosen between 0 and π. The sweep step of ρ and φ should be chosen to be small enough such that the required magnetic field accuracy is achieved. In this study, Δρ was chosen as 0.001 rad, which translates to ΔB≈ 1.9 mT (ΔH≈1.5 kA/m), and Δφ was chosen as 0.001 rad. It is worth noting that the analytical equation, i.e., Equation (19), involves a cosine function and is therefore insensitive to the sign of ρ, which changes according to the direction of magnetic field with reference to the fiber axis. Consequently, the technique, as such, is insensitive to the direction of the magnetic field.

The proposed distributed magnetic field measurement method demands a high degree of data smoothing, as local magnetic field measurement is resolved by locally fitting the filtered trace with the analytical equation Equation (20). Any unwanted local oscillations in the trace (due to noise) lead to measurement errors. In practice, the measured PSR, in our case ν-POTDR, traces are always affected by random noise, which originates from various sources [54]. Therefore, data smoothing is a prerequisite in quantifying the local magnetic field.

The Savitzky–Golay (SG) filtering technique [55,56] is quite effective when the underlying function can be locally well-fitted by a polynomial [57]. Such is the case of distributed magnetic field measurements performed in this study, as the analytical Equation (see Equation (19)) is a cosine function that can be efficiently fitted with a polynomial function. Two of the important filter parameters that dictate the effectiveness of the smoothing achieved by SG filtering are the filtering window (FW) and the order of the polynomial [58]. The amount of smoothing achieved increases with the FW and decreases as the polynomial order increases. However, larger FWs in general tend to distort the maxima and minima of the signal. Therefore, shorter FWs were employed in this study. However, as aforementioned, for the PSR trace-based distributed magnetic field measurement, a high degree of smoothing is required too, which is not possible with shorter FWs. In such cases, repeated filtering—passing the data to several SG filters back to back [59]—would be an effective solution [60,61]. In this study, for noise filtering of the experimental traces, we employed repeated SG filtering with polynomial order 2 and an FW that includes five data points.

## 3. Experimental Results

**Figure 3:**
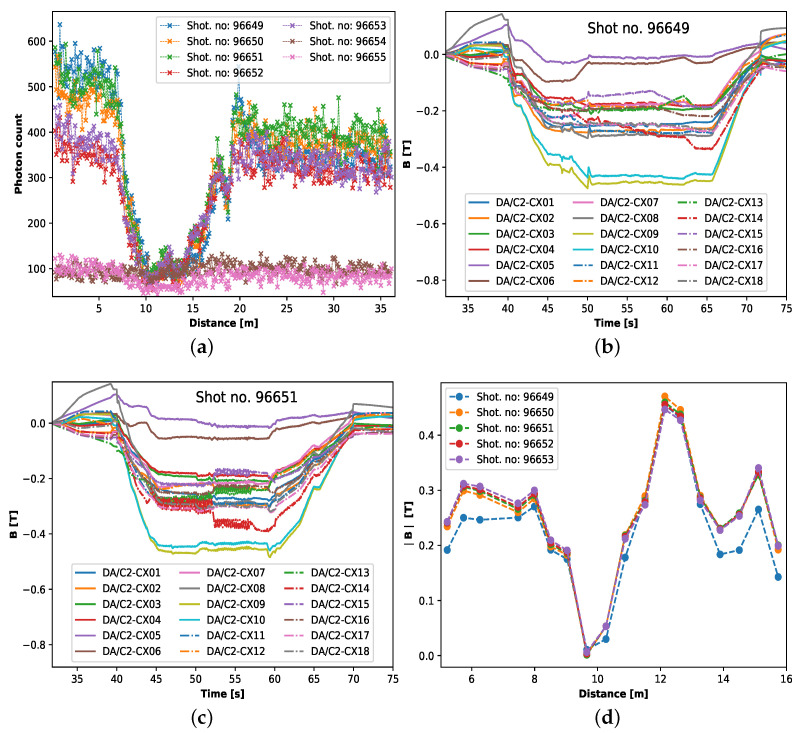
(**a**) Measured ν-POTDR traces for plasma shot no. 96649-55; (**b**) magnetic field measured by the 18 internal discrete coils for shot no. 96649 and (**c**) 96651; (**d**) absolute value of the magnetic field averaged over 10s for all the 18 internal discrete coils for shot no. 96649-53.

Figure 3a shows the ν-POTDR traces obtained from JET for the plasma shots 96649-55. During the plasma shots, each of the 18 internal discrete coils (IDCs), DA/C2 CX01-18, installed along the JET octant 3, where the sensing fiber is installed, measure a roughly constant magnetic field for a period of ∼15 s; see Figure 3b,c. During this period, a ν-POTDR measurement trace is recorded for each of the plasma shots, with pre-adjusted but different PC positions for each of the plasma shots. This is why we see a difference in the maximum photon count level for each of the measured traces; see Figure 3a. It is worth stressing here that in this proof-of-concept experiment, the choice of using different PC positions for each measurement trace (taken during each of the plasma shots) was made to verify the effectiveness of the measurement approach when the backscattered power level was not optimized, specifically when the maximum backscattered power level in the measurement trace was relatively close to the device noise floor. However, in practice, having one optimized PC position would have been sufficient for all the measurements, as the experimental arrangement remained relatively undisturbed throughout shots 96649-55, i.e., the Jones matrix corresponding to the optical path between the PC and the sensing fiber input is stable. As the photon count is proportional to the optical power [62], the ν-OTDR measured photon count corresponds to the Rayleigh backscattered power level. It is important to note that the difference in the measured traces from shot to shot is mostly due to different PC adjustments for each of the shots. Otherwise, all the measurement traces should look similar to one another, as the poloidal magnetic field generated during the shots is more or less similar; see Figure 3b,c, where two sets (for two different shots) of magnetic field measurements acquired by the IDCs are presented.

As the measurement traces taken for shots 96654 and 55 are buried under noise, we ignored them in further analysis. Figure 3d indicates the absolute value of the magnetic field induction, measured by the 18 IDCs, averaged over 10 s for the plasma shots of interest, i.e., shot no. 96649-53. The 10 s averaging of the magnetic field is due to the fact that the ν-POTDR measurement trace is taken over 10 s. Note that the distance scale in Figure 3d is obtained by calculating the position of these IDCs with reference to the sensing fiber around the JET VV section. It can be noticed from Figure 3d that the magnetic field generated during shot 96649 is slightly different from the other shots, i.e., shots 96650-53. Note that the IDC data for shot 96651 shown in Figure 3c are representative of shots 96650-53. Considering the similar appearance of the measured traces for plasma shots 96650-51 and 96652-53 (see Figure 3a), we averaged the mentioned trace pairs to improve the SNR. Therefore, we have three different traces for data analysis: trace obtained for shot no. 96649, the average of shots 96650 and 51 and the average of shots 96652 and 53.

**Figure 4:**
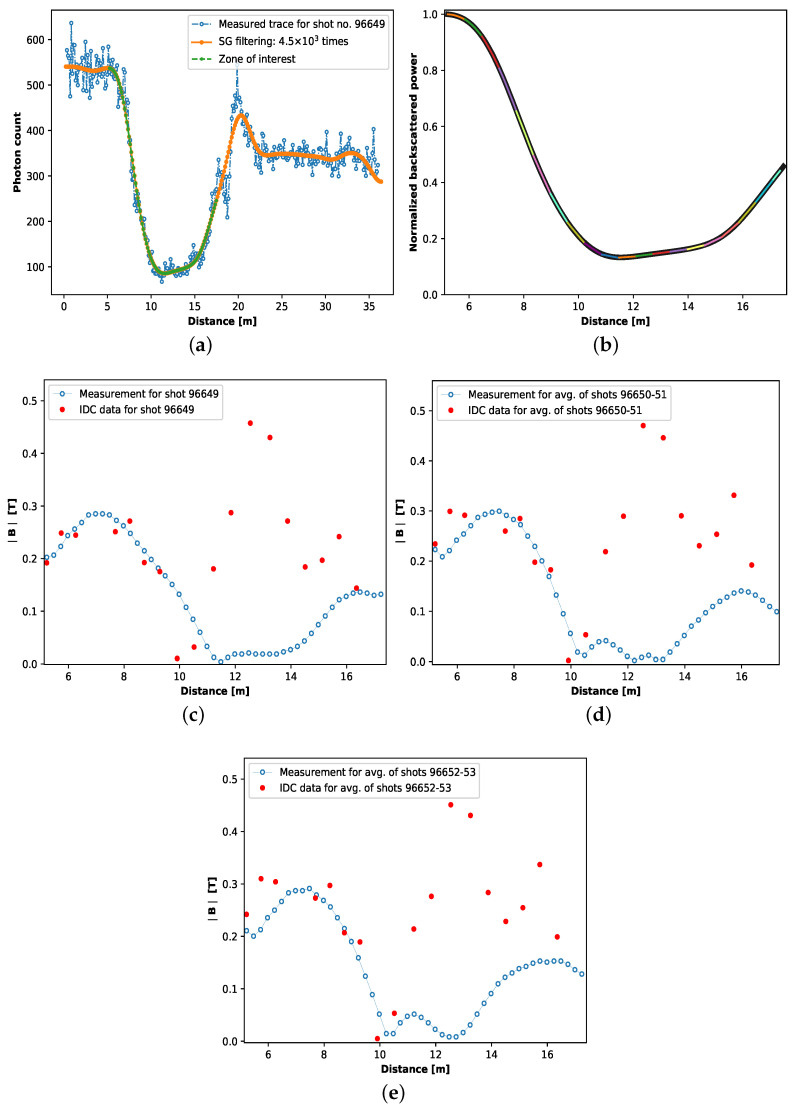
(**a**) Measured and filtered ν-POTDR trace for plasma shot no. 96649; (**b**) normalized measurement trace of shot no. 96649 within the sensing zone and fitted with local best fit using Equation (20); (**c**) comparison of the distributed poloidal magnetic field measured by the proposed ν-POTDR sensor and the internal discrete coil data for shot no. 96649, (**d**) average of shot no. 96650-51 and (**e**) average of shot no. 96652-53.

Figure 4a shows the measured trace and its noise-filtered version for plasma shot no. 96649, along with the zone of interest from which the distributed poloidal magnetic field measurement was deduced. Figure 4b shows the normalized measurement trace obtained for shot no. 96649 after filtering. Each of the colored sections corresponds to the local best-fitted $\rho_{i}$. In Figure 4c, the local poloidal magnetic field measured for plasma shot no. 96649 is presented along with the IDC data for the same shot. Recall that the local poloidal magnetic field measurements are based on the local best-fitted $\rho_{i}$ (see Equation (21)) obtained by using Equation (20) and sweeping the value of ρ and ϕ in the aforementioned range and sweep step. The measured magnetic field shows good agreement with the IDC data until around ∼11 m. It is rather important to note that the position of the IDCs and the optical fiber sensor on the VV section is such that there is a difference in the magnitude of the magnetic field experienced by the two sensors. Therefore, this is a qualitative analysis rather than a quantitative one. Furthermore, in general, accurate knowledge of the Verdet constant *V* of the sensing fiber is important for the accurate measurement of the magnetic field. In this study, we used a value of *V* calculated based on [26]. As seen in Figure 4a, after ∼11 m, up until ∼16 m, the ν-POTDR measurement is drawn into the noise. This region, ∼11–16 m, corresponds to the low backscatter power or photon count region in the measured trace, where the measured power reaches the noise floor of the device; see Figure 4a. It should be noted that the dynamic range (DR) of the device calculated from the trace reads 3.8 dB. Nevertheless, after ∼16 m, the measurement shows the sign of slow recovery; see Figure 4c. However, due to the limited sensing region available (less than 1 m) after 16 m, there is only one IDC measurement (and four optical fiber sensor measurement points) available to provide evidence of this recovery. Nevertheless, the results obtained from the region between 5 to 11 m allow us to say that when the measurement is not significantly affected by the device noise floor, we can extract the measurement. A potential solution to recover the magnetic field from the noise-prone region of the trace is discussed in Section 4. A similar result is observed for the magnetic field measured from the average of the traces obtained for shots 96650-51 and 96652-53, respectively, in Figure 4d,e. The spatial resolution of the reported magnetic field measurements is ∼26 cm, as local magnetic field measurements are made by fitting two data points; the spatial resolution of the ν-OTDR used in the experiments is ∼13 cm.

## 4. Approach for Avoiding the Issue Due to Reflectometer’s Noise Level

**Figure 5:**
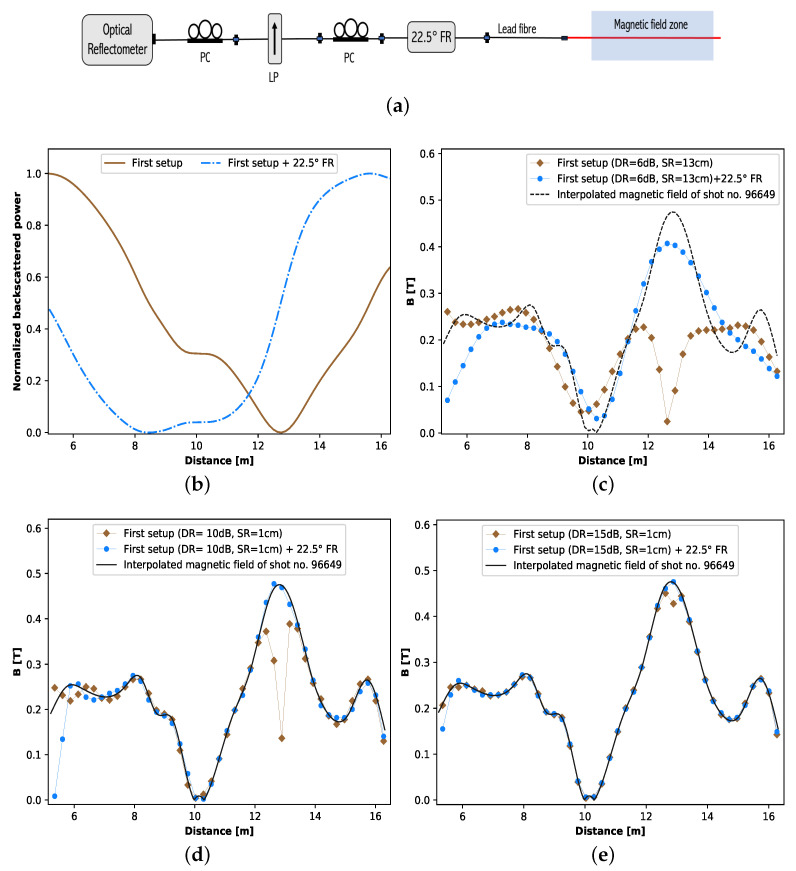
(**a**) Alternative ν-POTDR setup to recover the magnetic field measurement lost due to the reflectometer’s noise level; (**b**) simulated PSR trace without and with FR for interpolated IDC measured magnetic field from shot no. 96649; (**c**) comparison of the interpolated magnetic field used for the simulations with the magnetic field measured without and with FR for a PSR with 6 dB DR and 13 cm SR, (**d**) 10 dB DR and 1 cm SR and (**e**) 15 dB and 1 cm SR; DR: dynamic range, SR: sampling resolution, IDC: internal discrete coil.

The discussion on the experimental results has made it clear that the magnetic field information around the minima of the PSR trace can be corrupted when it reaches the noise floor of the device. A solution to this issue is to shift the minima of the PSR trace above the noise floor of the device. This task can be achieved in two ways. One way is to induce a known SOP rotation bias, so that the minima of the trace rise above the noise level. This solution facilities a way to use the relatively low DR PSRs, like ν-POTDRs, to determine the magnetic field profile. However, it comes at the cost of taking two measurements, one without the SOP rotation bias and the other with the SOP rotation bias. The schematic of the corresponding measurement setup is shown in Figure 5a. Note that the schematic of this alternative measurement setup is similar to the first method (see Figure 1), except for an extra PC and a 22.5° Faraday rotator (FR) placed after the LP. The role of the PC after the LP is to compensate for the effects in the lead fiber and help launch a desired SOP into the sensing fiber [63]. The 22.5° FR is used to rotate the SOP by 22.5° in both forward and backward propagation of the light, respectively, so that an extra 45° SOP rotation, compared to that of the First setup, is induced in the backscattered light reaching the detector. The purpose of this extra SOP rotation, as aforementioned, is to shift the minimum of the trace obtained with the first setup above the noise level. Particularly, the 45° extra SOP rotation facilitates to shift the minima of the trace to around 0.5 level of the normalized trace; this part of the trace is generally less susceptible to noise. The second approach is to use a PSR with a large enough DR that keeps the minima of the PSR trace well above the noise floor of the device. In this case, the experimental process remains the same as the one shown in Figure 1, but an optical reflectometer with a large DR, like an OFDR, has to be used in place of the ν-OTDR. However, it should be noted that this option comes with a higher equipment cost.

Due to the constraints of tight operating schedules and access limitations at JET, it was not feasible to verify the proposed methods experimentally on the JET tokamak. Therefore, the only viable option was to undertake a simulation approach to verify the proposed methods and justify their suitability for implementation on other tokamaks. The proposed methods can be simulated based on the sensor modeling presented in Section 2. When generating the simulated traces of the setup in Figure 5a, the Jones matrix corresponding to 22.5° FR has to be added in Equation (14) after $V_{in}$ and also before $M_{P}$. The Jones matrix corresponding to the required FR can be obtained by replacing $\theta_{i}$ with 22.5° in Equation (12). Using this sensor modeling, the simulated normalized PSR traces shown in Figure 5b are generated without (first setup) and with a 22.5° FR, considering the magnetic field profile obtained by interpolating (to match the spatial resolution of the considered PSR) the magnetic field measured by the IDCs for shot no. 96649. Figure 5c shows the distributed magnetic field measured from the simulated traces with the measurement configuration of the first setup (Figure 1) and the one with 22.5° FR (Figure 5a) for a PSR with a 6 dB DR and 13 cm spatial resolution. The figure indicates that the magnetic field measurement drawn into the noise, when using the first measurement setup, can be recovered by taking a second measurement by inserting an extra 22.5° FR after the polarizer. Note that the mentioned configuration of the PSR corresponds to a typical ν-POTDR. The effect of the DR on the normalized trace is considered as a Gaussian noise with zero mean and the standard deviation σ, where $\sigma =\frac{P_{B_{max}}}{10^{DR/5}}$ and $P_{B_{max}}$ is the maximum power in the linear scale, which for a normalized trace is 1 [64]. More details on the noise floor analysis of PSR traces can be found elsewhere [65]. It is worth stressing that the distributed magnetic field measurement results presented in Figure 5c are obtained after noise filtering the simulation traces generated for the mentioned PSR with the repeated SG filtering approach [60]. The desired number of SG filtering repeats is obtained from the number of filterings that have the LMSE [53] with the noise-free simulation trace, shown in Figure 5b. In this regard, the results in Figure 5c are a qualitative representation of the experimental results. It is worth emphasizing here that Figure 4b,c indeed provide evidence for the practical feasibility of this approach: On the one hand, the experimental results showed that the magnetic field was properly determined in the region around 8 m in Figure 4c, where the normalized backscattered power level was distributed around the 0.5 level; see Figure 4b. On the other hand, the inclusion of the 22.5° FR induces a shift in polarization rotation, causing the region of the trace that originally reached the low power level without the FR to move to the 0.5 level when the FR is employed. Therefore, it can be concluded that the 22.5° FR facilitated the retrieval of the magnetic field in the zone where the power level was initially too low.

In the other approach that can be used to avoid a part of the PSR trace being drawn into the noise floor, an optical reflectometer with a large dynamic range, like an OFDR, can be used in place of the ν-OTDR in Figure 1. In general, a typical OFDR has a DR of 10–15 dB and a sub-cm-range spatial resolution. Figure 5d shows the effectiveness of a PSR with 10 dB DR and a spatial resolution of 1 cm in determining the magnetic field profile. It can be clearly seen that the magnetic field profile is better determined in this case compared to that in Figure 5c. Nevertheless, by adding the 22.5° FR, even in this case, the magnetic field can be determined more accurately around the minima of the trace. However, this improvement is not significant compared to that seen in the 6 dB case in Figure 5c because, in Figure 5d, the magnetic field distribution is traced well along most of the fiber length even without 22.5° FR. Figure 5e shows the impact of high DR (15 dB) of the PSR in effectively determining the magnetic field profile. It should be noted that the spatial resolution of the magnetic field measurement in Figure 5c–e is maintained the same, i.e., 26 cm, to enable a fair comparison between the cases.

## 5. Conclusions

A novel method for measuring the distributed poloidal magnetic field in tokamaks using a polarization-sensitive reflectometric technique is proposed. The method was studied theoretically, and its feasibility was experimentally demonstrated using the ν-POTDR at JET tokamak. The experimental results show good qualitative agreement with the IDC data for the region of the PSR trace that is not corrupted by noise (high-SNR areas). Although the spatial resolution of the magnetic field measurement reported in this paper is ∼26 cm, the proposed technique when using an optical reflectometer such as an OFDR, with mm or sub-mm range spatial resolution, is capable of measuring the magnetic field with a cm or even sub-cm-range spatial resolution. An alternative measurement approach is proposed to address the issue of low SNR regions where significant differences between the optical fiber-based measurement and JET IDC measurement are observed. This approach is demonstrated through simulations considering the magnetic field profile measured by the JET IDCs for shot no. 96649. The simulation results indicate that to better determine the magnetic field profile, in general, a PSR with a large DR, more than 15 dB in the sensing region, is required. Nevertheless, if the DR of the PSR being used for magnetic field measurement is less than 15 dB, the magnetic field profile can still be determined by taking a second measurement with 22.5° FR inserted in the setup just before the lead fiber. This second measurement complements the first measurement taken without the FR in determining the magnetic field profile. 

## Data Availability

Data underlying the results presented in this paper are not publicly available.

## References

[1] Equipe T.F.R. (1978). Tokamak plasma diagnostics. Nucl. Fusion.

[2] Strait E.J. (2006). Magnetic diagnostic system of the DIII-D tokamak. Rev. Sci. Instrum..

[3] Biel W., Albanese R., Ambrosino R., Ariola M., Berkel M., Bolshakova I., Brunner K., Cavazzana R., Cecconello M., Conroy S. (2019). Diagnostics for plasma control–From ITER to DEMO. Fusion Eng. Des..

[4] Moreau P., Bolshakova I., Brichard B., Chitarin G., Delogu R., Duran I., Encheva A., Fournier Y., Galo A., Le-Luyer A. Development of a magnetic diagnostic suitable for the ITER radiation environment. Proceedings of the 2009 1st International Conference on Advancements in Nuclear Instrumentation, Measurement Methods and Their Applications.

[5] Vayakis G., Bertalot L., Encheva A., Walker C., Brichard B., Cheon M., Chitarin G., Hodgson E., Ingesson C., Ishikawa M. (2011). Nuclear technology aspects of ITER vessel-mounted diagnostics. J. Nucl. Mater..

[6] Girard S., Kuhnhenn J., Gusarov A., Brichard B., Van Uffelen M., Ouerdane Y., Boukenter A., Marcandella C. (2013). Radiation effects on silica-based optical fibers: Recent advances and future challenges. IEEE Trans. Nucl. Sci..

[7] Gusarov A., Leysen W., Wuilpart M., Mégret P. (2018). Status and future developments of R&D on fiber optics current sensor for ITER. Fusion Eng. Des..

[8] Gusarov A., Leysen W., Kim S.M., Dandu P., Wuilpart M., Danisi A., Vayakis G., Danisi A., Barbero J.L. (2023). Recent achievements in R&D on fibre optics current sensor for ITER. Fusion Eng. Des..

[9] Palmieri L., Schenato L. (2013). Distributed optical fiber sensing based on Rayleigh scattering. Open Opt. J..

[10] Aerssens M., Gusarov A., Moreau P., Malard P., Massaut V., Mégret P., Wuilpart M. Development of a Jones vector based model for the measurement of a plasma current in a thermonuclear fusion reactor with a POTDR setup. Proceedings of the Optical Sensing and Detection II.

[11] Wuilpart M., Aerssens M., Gusarov A., Moreau P., Mégret P. (2017). Plasma current measurement in thermonuclear fusion reactors using a photon-counting POTDR. IEEE Photonics Technol. Lett..

[12] Ross J. (1981). Measurement of magnetic field by polarisation optical time-domain reflectometry. Electron. Lett..

[13] Rogers A.J. (1981). Polarization-optical time domain reflectometry: A technique for the measurement of field distributions. Appl. Opt..

[14] Galtarossa A., Palmieri L. Mapping of intense magnetic fields based on polarization sensitive reflectometry in single mode optical fibers. Proceedings of the 2013 Africon, Pointe aux Piments.

[15] Palmieri L., Galtarossa A. Reflectometric fiber optic sensor for distributed measurement of intense magneto-static fields. Proceedings of the SENSORS, 2011 IEEE.

[16] Laming R.I., Payne D.N. (1989). Electric current sensors employing spun highly birefringent optical fibers. J. Light. Technol..

[17] Wegmuller M., Scholder F., Gisin N. (2004). Photon-counting OTDR for local birefringence and fault analysis in the metro environment. J. Light. Technol..

[18] Masoudi A., Newson T.P. (2014). Distributed optical fiber dynamic magnetic field sensor based on magnetostriction. Appl. Opt..

[19] Ding Z., Du Y., Liu T., Liu K., Feng B., Jiang J. (2015). Distributed optical fiber current sensor based on magnetostriction in OFDR. IEEE Photonics Technol. Lett..

[20] Veeser L.R., Chandler G.I., Day G.W. Fiber optic sensing of pulsed currents. Proceedings of the Photonics: High Bandwidth Analog Applications, Proceedings of the SPIE.

[21] Rappé G. (1977). The JET (joint European torus) vacuum vessel. Rev. De Phys. Appl..

[22] Buckingham A., Stephens P. (1966). Magnetic optical activity. Annu. Rev. Phys. Chem..

[23] Haider T. (2017). A review of magneto-optic effects and its application. Int. J. Electromagn. Appl..

[24] Piller H. (1972). Faraday rotation. Semiconductors and Semimetals.

[25] Kim B., Park D., Choi S. (1982). Use of polarization-optical time domain reflectometry for observation of the Faraday effect in single-mode fibers. IEEE J. Quantum Electron..

[26] Cruz J., Andres M., Hernandez M. (1996). Faraday effect in standard optical fibers: Dispersion of the effective Verdet constant. Appl. Opt..

[27] Faraday M. (1846). XXVII. On the magnetic affection of light, and on the distinction between the ferromagnetic and diamagnetic conditions of matter. Lond. Edinb. Dublin Philos. Mag. J. Sci..

[28] Palmieri L., Galtarossa A. (2011). Distributed polarization-sensitive reflectometry in nonreciprocal single-mode optical fibers. J. Light. Technol..

[29] Noda J., Hosaka T., Sasaki Y., Ulrich R. (1984). Dispersion of Verdet constant in stress-birefringent silica fibre. Electron. Lett..

[30] Rose A., Etzel S.M., Wang C.M. (1997). Verdet constant dispersion in annealed optical fiber current sensors. J. Light. Technol..

[31] Simon A., Ulrich R. (1977). Evolution of polarization along a single-mode fiber. Appl. Phys. Lett..

[32] Chen H.c., Wen J.x., Huang Y., Dong W.l., Pang F.f., Luo Y.h., Peng G.d., Chen Z.y., Wang T.y. (2017). Influence of linear birefringence on Faraday effect measurement for optical fibers. Optoelectron. Lett..

[33] Aerssens M., Descamps F., Gusarov A., Mégret P., Moreau P., Wuilpart M. (2015). Influence of the optical fiber type on the performances of fiber-optics current sensor dedicated to plasma current measurement in ITER. Appl. Opt..

[34] Rashleigh S., Ulrich R. (1979). Magneto-optic current sensing with birefringent fibers. Appl. Phys. Lett..

[35] Barlow A., Ramskov-Hansen J., Payne D. (1981). Birefringence and polarization mode-dispersion in spun single-mode fibers. Appl. Opt..

[36] Li L., Qian J., Payne D.N. (1987). Miniature multi-turn fibre current sensors. Int. J. Opt. Sens..

[37] Li L., Qian J.R., Payne D. (1986). Current sensors using highly birefringent bow-tie fibres. Electron. Lett..

[38] Payne D.N., Barlow A.J., Hansen J.R. (1982). Development of low-and high-birefringence optical fibers. IEEE Trans. Microw. Theory Tech..

[39] Barlow A., Ramskov-Hansen J., Payne D.N. (1982). Anisotropy in spun single-mode fibres. Electron. Lett..

[40] Przhiyalkovsky Y.V., Vasiliev S., Medvedkov O., Morshnev S., Dianov E. (2017). Polarization state evolution in spun birefringent optical fibers. J. Appl. Phys..

[41] Morshnev S.K., Gubin V.P., Vorob’ev I., Starostin I., Sazonov A.I., Chamorovsky Y.K., Korotkov N. (2009). Spun optical fibres: A helical structure of linear birefringence or circular birefringence?. Quantum Electron..

[42] Kim S.M., Dandu P., Gusarov A., Danisi A., Vayakis G., Wuilpart M. (2023). Assessment of the Structural Vibration Effect on Plasma Current Measurement Using a Fiber Optic Current Sensor in ITER. Sensors.

[43] Dandu P., Goussarov A., Moreau P., Leysen W., Kim S., Mégret P., Wuilpart M. Polarization-OTDR-based optical fibre sensor for plasma current measurement in ITER: Effect of fibre bending, twisting and temperature dependence of Verdet constant on the measurement accuracy. Proceedings of the Optical Sensors 2021.

[44] Jones R.C. (1941). A new calculus for the treatment of optical systems I. Description and discussion of the calculus. Josa.

[45] Hurwitz H., Jones R. (1941). A new calculus for the treatment of optical systems II. Proof of three general equivalence theorems. J. Opt. Soc. Am..

[46] Jones R.C. (1948). A new calculus for the treatment of optical systems. VII. Properties of the N-matrices. Josa.

[47] Dandu P., Gusarov A., Moreau P., Leysen W., Kim S., Mégret P., Wuilpart M. (2022). Plasma current measurement in ITER with a polarization-OTDR: Impact of fiber bending and twisting on the measurement accuracy. Appl. Opt..

[48] Rogers A., Zhou Y., Handerek V. Computational polarization-optical time domain reflectometry for measurement of the spatial distribution of PMD in optical fibers. Proceedings of the 4th Optical Fiber Measurement Conference.

[49] Ross J.N. (1982). Birefringence measurement in optical fibers by polarization-optical time-domain reflectometry. Appl. Opt..

[50] Wuilpart M. (2011). Rayleigh scattering in optical fibers and applications to distributed measurements. Advanced Fiber Optics: Concepts and Technology.

[51] Pistoni N.C. (1995). Simplified approach to the Jones calculus in retracing optical circuits. Appl. Opt..

[52] VanWiggeren G.D., Roy R. (1999). Transmission of linearly polarized light through a single-mode fiber with random fluctuations of birefringence. Appl. Opt..

[53] Taylor J. (1997). Introduction to Error Analysis, the Study of Uncertainties in Physical Measurements.

[54] Healey P. (1984). Optical time domain reflectometry—a performance comparison of the analogue and photon counting techniques. Opt. Quantum Electron..

[55] Savitzky A., Golay M.J. (1964). Smoothing and differentiation of data by simplified least squares procedures. Anal. Chem..

[56] Schafer R.W. (2011). What is a Savitzky-Golay filter?[lecture notes]. IEEE Signal Process. Mag..

[57] Press W.H., Teukolsky S.A. (1990). Savitzky-Golay smoothing filters. Comput. Phys..

[58] King R.L., Ruffin C., LaMastus F., Shaw D. The analysis of hyperspectral data using Savitzky-Golay filtering-practical issues. 2. Proceedings of the IEEE 1999 International Geoscience and Remote Sensing Symposium. IGARSS’99 (Cat. No. 99CH36293).

[59] Chan S., Leong L. (1972). Analysis of least squares smoothing operators in the frequency domain. Geophys. Prospect..

[60] Proctor A., Sherwood P.M. (1980). Smoothing of digital X-ray photoelectron spectra by an extended sliding least-squares approach. Anal. Chem..

[61] Bromba M.U., Ziegler H. (1981). Application hints for Savitzky-Golay digital smoothing filters. Anal. Chem..

[62] Hecht E. (2017). Electromagnetic Theory, Photons, and Light. Proceedings of the Optics.

[63] Grexa M., Hermann G., Lasnitschka G., Scharmann A. (1984). Faraday rotation in a single-mode fiber with controlled birefringence. Appl. Phys. B.

[64] Anderson D.R., Bell F.G. (1997). Optical Time-Domain Reflectometry.

[65] Motuz R., Leysen W., Moreau P., Gusarov A., Drexler P., Wuilpart M. (2019). Theoretical assessment of the OTDR detector noise on plasma current measurement in tokamaks. Appl. Opt..

